# Dexmedetomidine Ameliorates Lung Injury Induced by Intestinal Ischemia/Reperfusion by Upregulating Cannabinoid Receptor 2, Followed by the Activation of the Phosphatidylinositol 3-Kinase/Akt Pathway

**DOI:** 10.1155/2020/6120194

**Published:** 2020-06-21

**Authors:** Meng Chen, Xue-Tao Yan, Li Ye, Jun-Jiao Tang, Zong-Ze Zhang, Xiang-Hu He

**Affiliations:** ^1^Department of Anesthesiology, Zhongnan Hospital of Wuhan University, Wuhan, Hubei 430071, China; ^2^Department of Anesthesiology, Shenzhen Bao'an Maternity and Child Health hospital, Shenzhen 518133, China

## Abstract

Intestinal ischemia/reperfusion (I/R) is a clinical emergency, which often causes lung injury with high morbidity and mortality. Although dexmedetomidine has been identified to have a protective effect on lung injury caused by intestinal I/R, its specific mechanism is still elucidated. In recent years, the cannabinoid (CB_2_) receptor pathway has been found to be involved in I/R injury of some organs. In the current study, we investigated whether the CB_2_ receptor pathway contributes to the protective effect of dexmedetomidine on the intestinal I/R-induced lung injury in rats. Dexmedetomidine treatment upregulated the expression of CB_2_ receptor and suppressed the I/R-induced increases in lung injury scores, inflammatory cell infiltration, lung wet/dry ratio, MPO activity, MDA level, inflammatory cytokines, and caspase-3 expression while augmenting SOD activity and Bcl-2 expression, indicating attenuation of lung injury. Dexmedetomidine treatment also increased the expression of Akt. The protective effects of dexmedetomidine treatment were reversed by the CB_2_ receptor antagonist AM630 or the PI3K inhibitor wortmannin. And the CB_2_ receptor antagonist AM630 also downregulated the expression of Akt. Thus, our findings suggest that treatment with dexmedetomidine provides a protective role against lung injury caused by intestinal I/R in rats, possibly due to the upregulation of the CB_2_ receptor, followed by the activation of the PI3K/Akt pathway.

## 1. Introduction

Intestinal ischemia/reperfusion (I/R) is a clinical emergency frequently occurring in multiple clinical conditions, including acute mesenteric ischemia, abdominal aortic aneurysm surgery, small bowel transplantation, shock, and cardiopulmonary bypass [1]. In addition to causing local intestinal damage, I/R is often followed by distant organ injury, especially lung injury, associated with high morbidity and mortality [2]. Although the exact mechanism is poorly understood, some factors, such as oxidative stress, activated neutrophils, complement components, released inflammatory cytokines, and cell apoptosis, are believed to be involved in the development of I/R-induced lung injury [3–5].

Dexmedetomidine (DEX), a highly selective alpha-2 adrenoceptor (*α*_2_AR) agonist, exhibits anesthetic-sparing, analgesia, and sympatholytic properties and is widely used as a sedative agent in clinical anesthesia and ICU management [6]. Previous studies have identified DEX as having protective effects in some models of I/R injury, such as in the liver [7, 8], lung [9], heart [10], kidney [11], and intestine [12] because of its antioxidant, anti-inflammatory, and antiapoptotic properties. Further research has shown that some molecular mechanisms, such as *α*_2_AR/phosphatidylinositol 3-kinase (PI3K)/Akt [13], HMGB1-TLR_4_-MyD88-NF-*к*B [14], and JAK/STAT [15], are involved in the process of protection of DEX against some organ I/R injuries. It has also been demonstrated that treatment with DEX mitigated lung injury caused by various factors, including intestinal I/R [16]. Although the protective effect of DEX treatment on intestinal I/R-induced lung injury has been investigated, the potential molecular mechanism by which DEX ameliorates lung injury remains unclear.

Recently, the role of cannabinoid (CB) receptors, especially the CB_2_ receptor, has attracted much attention in health and disease. Although the CB_2_ receptor was first identified in the peripheral immune system, it is now expressed in most organ systems, including in the cells of the brain, heart, liver, and cardiovascular and gastrointestinal (GI) systems [17]. Early studies focused mainly on the role of the CB_2_ receptor in fundamental physiological processes. However, accumulating evidence supports that the CB_2_ receptor has a critical role in regulating many disease processes, including inflammation [18], cancer [19], pain [20], and I/R injury [21], with it shown that the CB_2_ agonist, HU-910, protected against hepatic I/R injury by attenuating oxidative stress, inflammatory response, and apoptosis [22]. Using the selective CB_2_ agonist, JWH-133, and CB_2_ gene-deficient mice, Defer et al. [23] also revealed that activating the CB_2_ receptor promoted the survival of cardiac myocytes and protected against I/R-induced myocardial infarction through the direct inhibition of myocyte and fibroblast death and the prevention of myofibroblast activation. The role of the CB_2_ receptor in the GI tract has been investigated, including in regulating abnormal motility, modulating intestinal inflammation, and limiting visceral sensitivity and pain [18, 24]. However, it remains unclear whether the CB_2_ receptor is involved in the intestinal I/R-induced lung injury.

Thus, we hypothesized that DEX might improve intestinal I/R-induced lung injury by activating the CB_2_ receptor pathway. In this study, we established a rat model of lung injury induced by intestinal I/R and investigated the effect of DEX on intestinal I/R-induced lung injury and its potential mechanisms.

## 2. Materials and Methods

### 2.1. Animals

Male Sprague-Dawley rats weighing 220-270 g were obtained from the Department of Laboratory Animal Center of the Wuhan University and housed under standardized conditions of food, water, light, and temperature. All animals were subjected to fasting for 12 h before experiments, but they were allowed free access to tap water throughout the experimental procedure. All procedures were approved by the Research Committee on Ethics of the Zhongnan Hospital of Wuhan University.

### 2.2. Drugs

DEX was acquired from Jiangsu Nhwa Pharmaceutical Corporation Ltd. (Jiangsu, China). The CB_2_R antagonist AM630 was obtained from Tocris (Baldwin, MO, USA). The PI3K inhibitor wortmannin was purchased from Sigma-Aldrich (St. Louis, MO, USA). AM630 and wortmannin were dissolved in DMSO. DMSO alone was used in control experiments.

### 2.3. Experimental Protocol

Fifty-six male Sprague-Dawley rats were randomly assigned to the following seven groups (*n* = 8): sham operation (Sham) group, intestinal I/R (I/R) group, dexmedetomidine-treated I/R (DEX) group, dexmedetomidine- and AM630-treated I/R (DA) group, AM630-treated I/R (AI) group, dexmedetomidine- and wortmannin-treated I/R (DW) group, and wortmannin-treated I/R (WI) group. Animals in the DEX, DA, and DW groups were infused continuously with dexmedetomidine (i.v.) at 5 *μ*g kg^−1^ h^−1^ for 1 h before ischemia. This dose was chosen based on the results from a previous study [12]. Animals in the DA, AI, DW, and WI groups were given the CB_2_ receptor antagonist, AM630 (3 mg/kg, i.p.), or the PI3K inhibitor, wortmannin (15 *μ*g/kg, i.v.), before the injection of dexmedetomidine. Animals in the Sham or I/R groups were given an equal volume of the vehicle at the same time point.

### 2.4. Surgical Procedure

The model of intestinal I/R injury was established as we previously described [25]. Each animal was anesthetized with an intraperitoneal injection of sodium pentobarbital (50 mg/kg). An upper midline laparotomy was performed to expose the abdomen and the superior mesenteric artery (SMA). An atraumatic microvascular clamp was applied to the SMA for 1 h of ischemia induction, after which the occluding clamp was removed for 2 h of reperfusion. Sham-operated animals underwent the same surgical procedure as the experimental rats, except for the induction of I/R injury. The animals were sacrificed with pentobarbital overdose, and lung samples were harvested after reperfusion and used to assess biochemical parameters or fixed in buffered formalin for histopathological evaluation.

### 2.5. Histological Assessment

Lung samples were immersed in 10% formalin and then embedded in paraffin. Tissue blocks were cut into 5 *μ*m sections and stained with hematoxylin and eosin (HE). The morphologic examination of lung tissues was carried out under a light microscope by two pathologists blinded to the experiment. The histological injury was graded using our previously described method [26]: grade 0, no diagnostic change; grade 1, mild neutrophil infiltration and mild to moderate interstitial congestion; grade 2, moderate neutrophil infiltration, perivascular edema formation, and partial destruction of the pulmonary architecture; and grade 3, dense neutrophil infiltration and the complete destruction of the pulmonary architecture.

### 2.6. Assessment of Pulmonary Edema

The left lower lung lobe was removed at the end of reperfusion, weighed, and then dried in an oven at 80°C for 24 h. The wet weight to dry weight (*W*/*D*) ratio of the lung tissue was calculated and used as an indicator for estimating pulmonary edema.

### 2.7. Assay of Myeloperoxidase Activity

Frozen lung tissues were homogenized on ice using a homogenizer. The myeloperoxidase (MPO) activity was measured according to the manufacturer's instructions (Jiancheng Biologic Project Company, Nanjing, China). One MPO activity unit was defined as the amount of lung tissue that converted 1 *μ*mol of hydrogen peroxide to water per minute at 37°C. The MPO activity was expressed in units per gram (U/g) of wet weight.

### 2.8. Assay of Lipid Peroxidation and Superoxide Dismutase Activity

Frozen lung tissues were homogenized on ice using a homogenizer and centrifuged at 4000 g for 10 min at 4°C. Malondialdehyde (MDA) level and superoxide dismutase (SOD) activity in the supernatant were determined according to the manufacturer's instructions using commercial kits (Jiancheng Bioengineering Institute, Nanjing, China). The SOD activity was expressed in U/mg protein, and the MDA level was expressed in nmol/mg protein.

### 2.9. Assay of Lung Tissue Cytokines

Frozen lung tissues were homogenized on ice using a homogenizer and centrifuged at 4000 g for 10 min at 4°C. Levels of tumor necrosis factor alpha (TNF-*α*) and interleukin 6 (IL-6) in the supernatant were assayed according to the manufacturer's instructions using ELISA kits (R&D Systems, Minneapolis, MN, USA). The concentrations of TNF-*α* and IL-6 were expressed in pg/mg protein.

### 2.10. Immunohistochemistry

Immunohistochemistry was performed to detect macrophage infiltration and phosphorylated Akt (p-Akt) expression in the lung tissues. F4/80 is a marker of macrophages, and its expression is used to detect macrophage infiltration. Sections were incubated with 5% bovine serum albumin and then with primary antibodies: F4/80 (Thermo Fisher Scientific, USA) and p-Akt (Cell Signaling Technology, USA). The sections were incubated in species-specific secondary antibodies labeled with horseradish peroxidase and then visualized by incubating the sections with DAB (Boster Bioengineering, Wuhan, China). Expressions of F4/80 and p-Akt were quantified by measuring the integrated optical density (IOD) of the positive staining area.

### 2.11. Real-Time Quantitative Reverse Transcription-PCR

The total RNA of rat lung tissues was extracted using TRIzol reagent according to the manufacturer's instructions (Takara, Japan). A real-time quantitative reverse transcription-PCR (RT-PCR) analysis was performed by using a SYBR Premix Ex Taq™ Kit (Takara, Japan), and the reactions were conducted on a StepOne™ Real-Time PCR instrument (Life Technologies, Grand Island, NY). The primers used for PCR were as follows: caspase-3 forward 5′-actactgccggagtctgact-3′; reverse 5′-taaccgggtgcggtagagta-3′; Bax forward 5′-gaaccatcatgggctggaca-3′; reverse 5′-gtgagtgaggcagtgaggac-3′; Bcl-2 forward 5′-cttctctcgtcgctaccgtc-3′; reverse 5′-ggggtgacatctccctgttg-3′; Akt forward 5′-gagaaccgtgtcctgcagaa-3′; reverse 5′-gttctccagcttgaggtccc-3′; and GAPDH forward 5′-tgatgggtgtgaaccacgag-3′; reverse 5′-agtgatggcatggactgtgg-3′. PCR conditions were as follows: 95°C for 5 min; 35 cycles at 95°C for 20s, 60°C for 20s, and 72°C for 45 s. GAPDH was selected as an internal control, and the target gene expression was normalized to GAPDH expression and calculated using the 2^−*ΔΔ*Ct^ method.

### 2.12. Western Blot Analysis

Frozen lung samples were homogenized and centrifuged at 12,000 g for 10 min at 4°C. The protein concentrations in the supernatant were measured using a BCA protein assay kit (Beyotime Institute of Biotechnology, Shanghai, China). Equal amounts of lysate were subjected to 10% sodium dodecyl sulfate-polyacrylamide gel electrophoresis, then transferred to polyvinylidene fluoride membranes (Millipore, Bedford, MA, USA) that were incubated with primary antibodies against cleaved caspase-3, Bax, Bcl-2 (Santa Cruz Biotechnology, USA), and p-Akt (Cell Signaling Technology, USA) overnight at 4°C. The membranes were washed with PBS-0.05% Tween-20 the next day and incubated for 1 h in horseradish peroxidase-conjugated secondary antibody (Jackson ImmunoResearch). Another wash of the membranes with PBS-0.05% Tween 20 followed the 1 h incubation period, at the end of which protein bands were visualized using an enhanced chemiluminescence kit (Amersham, Piscataway, NJ, USA). All band densities were quantified by densitometry using the Quantity One software (BioRad, Hercules, CA, USA).

### 2.13. Statistical Analysis

All data are expressed as mean values ± SEM. Differences among the groups were analyzed using the one-way analysis of variance (ANOVA), followed by the Student-Newman-Keul (SNK) test for multiple comparisons. Differences were considered significant at *P* < 0.05.

## 3. Results

### 3.1. The Effects of Dexmedetomidine Treatment on the Histological Injury

**Figure 1:**
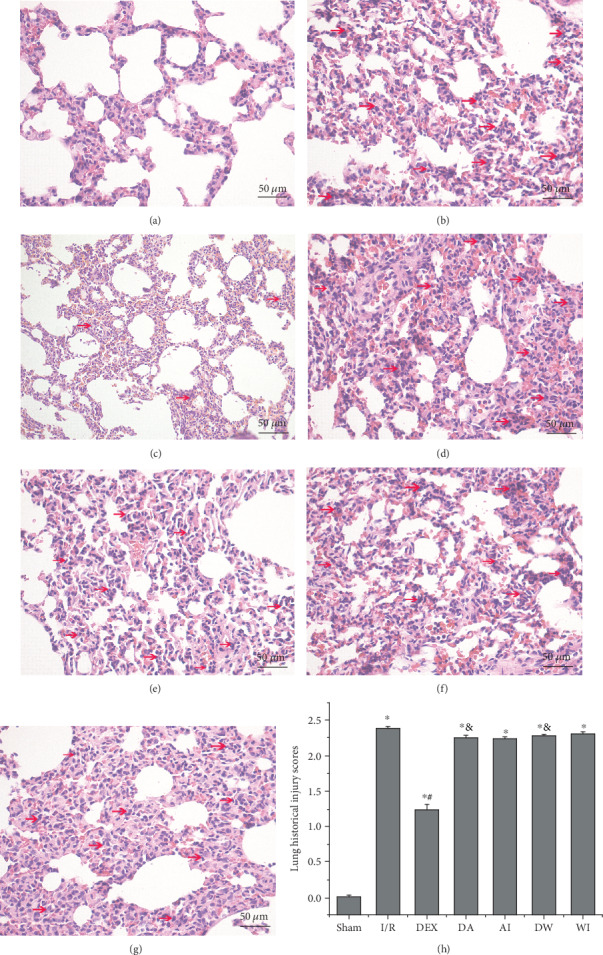
Histological changes and histological injury scores in lung tissues of all groups. (a) Sham group. (b) Intestinal ischemia/reperfusion (I/R) group. (c) Dexmedetomidine-treated I/R (DEX) group. (d) Dexmedetomidine- and AM630-treated I/R (DA) group. (e) AM630-treated I/R (AI) group. (f) Dexmedetomidine- and wortmannin-treated I/R (DW) group. (g) Wortmannin-treated I/R (WI) group. Red arrows indicate neutrophil infiltration (original magnification, ×200; scale bar = 50 *μ*m). (h) Histological injury scores of the lung. Data are expressed as mean ± SEM, *n* = 8 rats per group. ^∗^*P* < 0.05 versus the Sham group, ^#^*P* < 0.05 versus the I/R group, ^&^*P* < 0.05 versus the DEX group.

Lung histological injury was evaluated and scored by two pathologists to investigate the role of DEX treatment against lung injury caused by intestinal I/R; representative morphological changes are presented in Figure 1. No significant morphological changes were observed in the Sham group (Figure 1(a)). I/R induced obvious lung tissue injury, manifested by the apparent destruction of the pulmonary architecture, interstitial edema, hemorrhage, and massive neutrophil infiltration (Figure 1(b)). The use of DEX attenuated lung injury significantly, with mild neutrophil infiltration and interstitial congestion (Figure 1(c)). Significant morphological changes were observed in the other four groups (Figures 1(d)–1(g)). The average scores were used for analysis (Figure 1(h)). These data showed that the lung injury scores in the I/R group were higher than those in the Sham group (*P* < 0.05) and were attenuated by DEX treatment in the DEX group (*P* < 0.05) but not in the other four groups (*P* > 0.05).

### 3.2. The Effects of Dexmedetomidine Treatment on Lung Tissue *W*/*D* Weight Ratio

**Figure 2:**
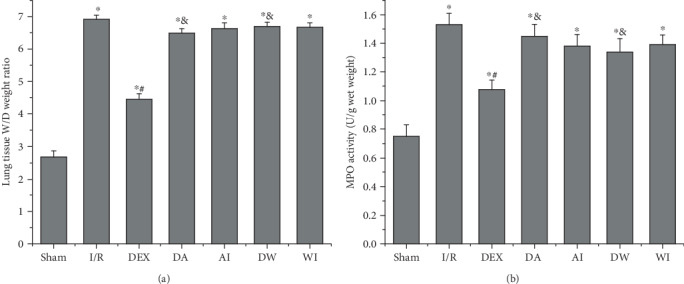
The effects of dexmedetomidine treatment on wet/dry (*W*/*D*) weight ratio and myeloperoxidase (MPO) activity in lung tissues. (a) *W*/*D* weight ratio. (b) MPO activity. Data are expressed as mean ± SEM, *n* = 8 rats per group. ^∗^*P* < 0.05 versus the Sham group, ^#^*P* < 0.05 versus the I/R group, ^&^*P* < 0.05 versus the DEX group. I/R: intestinal ischemia/reperfusion; DEX: dexmedetomidine-treated I/R; DA: dexmedetomidine- and AM630-treated I/R; AI: AM630-treated I/R; DW: dexmedetomidine- and wortmannin-treated I/R; WI: wortmannin-treated I/R.

The *W*/*D* weight ratio is used as an important indicator of lung tissue damage. As presented in Figure 2(a), the lung tissue *W*/*D* weight ratio was higher in all groups that underwent intestinal I/R than in the Sham group (*P* < 0.05). DEX treatment reduced the lung tissue *W*/*D* weight ratio compared to that of the I/R rats (*P* < 0.05). However, the protective role of DEX treatment against lung injury caused by intestinal I/R was reversed by the administration of the CB_2_ receptor antagonist, AM630, or the PI3K inhibitor, wortmannin (*P* < 0.05). There was no significant difference in the lung tissue *W*/*D* weight ratio in the animals treated with AM630 alone or wortmannin alone compared with animals in the I/R group (*P* > 0.05).

### 3.3. The Effects of Dexmedetomidine Treatment on MPO Activity

MPO activity is an indicator of neutrophil infiltration. We assessed the MPO activity to investigate the role of neutrophil infiltration in intestinal I/R-induced lung injury. As shown in Figure 2(b), I/R increased the MPO activity in lung tissues compared to that of the sham-operating rats (*P* < 0.05). Treatment with DEX decreased the MPO activity in lung tissues compared to that of the I/R rats (*P* < 0.05). In contrast, treatment with both AM630 and wortmannin reversed the decrease in MPO activity compared to that of the DEX rats (*P* < 0.05). Treatment with AM630 alone or wortmannin alone had no significant effect on MPO activity compared to that of the I/R rats (*P* > 0.05).

### 3.4. The Effects of Dexmedetomidine Treatment on SOD Activity and MDA Level

**Figure 3:**
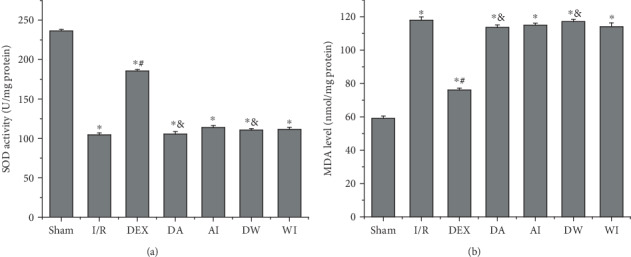
The effects of dexmedetomidine treatment on superoxide dismutase (SOD) activity and malondialdehyde (MDA) level in lung tissues. (a) SOD activity. (b) MDA level. Data are expressed as mean ± SEM, *n* = 8 rats per group. ^∗^*P* < 0.05 versus the Sham group, ^#^*P* < 0.05 versus I/R group, ^&^*P* < 0.05 versus DEX group. I/R: intestinal ischemia/reperfusion; DEX: dexmedetomidine-treated I/R; DA: dexmedetomidine- and AM630-treated I/R; AI: AM630-treated I/R; DW: dexmedetomidine- and wortmannin-treated I/R; WI, wortmannin-treated I/R.

Oxidative stress is one of the main causes of lung injury induced by intestinal I/R. We assessed the level of MDA, an indicator of lipid peroxidation damage, and the activity of SOD, an antioxidant enzyme, to examine the role of oxidative stress in lung injury. As shown in Figure 3, I/R increased the MDA level and decreased the SOD activity compared to those of the sham-operating rats (*P* < 0.05). DEX treatment led to a decrease in MDA level and an increase in SOD activity (*P* < 0.05). However, the protective effect of DEX treatment on lung injury was reversed by the application of AM630 or wortmannin (*P* < 0.05). Treatment with AM630 alone or wortmannin alone had no significant impact on MDA level and SOD activity compared to those of the I/R rats (*P* > 0.05).

### 3.5. The Effects of Dexmedetomidine Treatment on the Levels of TNF-*α* and IL-6

**Figure 4:**
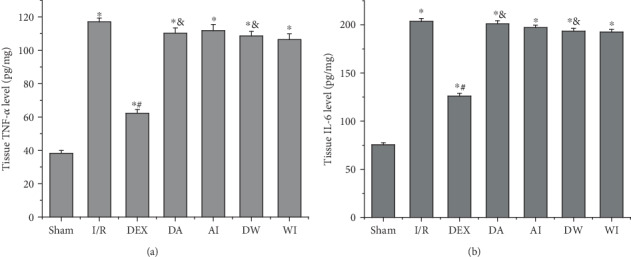
The effects of dexmedetomidine treatment on the levels of tumor necrosis factor alpha (TNF-*α*) and interleukin 6 (IL-6) in the lung tissues of all groups. (a) TNF-*α* level. (b) IL-6 level. Data are expressed as mean ± SEM, *n* = 8 rats per group. ^∗^*P* < 0.05 versus the Sham group, ^#^*P* < 0.05 versus the I/R group, ^&^*P* < 0.05 versus the DEX group. I/R: intestinal ischemia/reperfusion; DEX: dexmedetomidine-treated I/R; DA: dexmedetomidine- and AM630-treated I/R; AI: AM630-treated I/R; DW: dexmedetomidine- and wortmannin-treated I/R; WI: wortmannin-treated I/R.

We assessed the levels of TNF-*α* and IL-6 in the lung to evaluate the inflammatory cytokines in the lung injury caused by intestinal I/R. As shown in Figure 4, I/R led to an increase in the levels of TNF-*α* and IL-6 compared to those of the sham-operating rats (*P* < 0.05). Treatment with DEX decreased the levels of TNF-*α* and IL-6 compared to those of the I/R rats (*P* < 0.05). In contrast, treatment with AM630 or wortmannin reversed the protective role of DEX by increasing the levels of TNF-*α* and IL-6 (*P* < 0.05). AM630 alone or wortmannin alone had no significant impact on the levels of TNF-*α* and IL-6 compared to those of the I/R rats (*P* > 0.05). These results suggest that DEX treatment has a protective role against I/R-induced lung injury by reducing cytokine production.

### 3.6. The Effects of Dexmedetomidine Treatment on Macrophage Infiltration and p-Akt Expression in Lung Tissue by Immunohistochemical Assay

**Figure 5:**
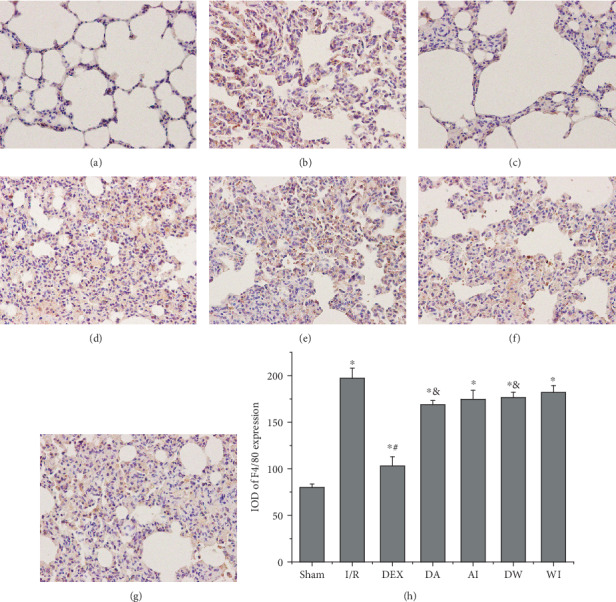
Immunohistochemical analysis of macrophage infiltration in the lung tissues of all groups. Macrophage infiltration was investigated by detecting F4/80 expression with immunohistochemistry. Dexmedetomidine treatment reduced macrophage infiltration in the lung tissues following I/R. (a) Sham group. (b) Intestinal ischemia/reperfusion (I/R) group. (c) Dexmedetomidine-treated I/R (DEX) group. (d) Dexmedetomidine- and AM630-treated I/R (DA) group. (e) AM630-treated I/R (AI) group. (f) Dexmedetomidine- and wortmannin-treated I/R (DW) group. (g) Wortmannin-treated I/R (WI) group (original magnification, ×400). (h) Integrated optical density (IOD) of F4/80 expression in all groups. Data are expressed as mean ± SEM, *n* = 6 rats per group. ^∗^*P* < 0.05 versus the Sham group, ^#^*P* < 0.05 versus the I/R group, ^&^*P* < 0.05 versus the DEX group.

To assess macrophage infiltration in the lungs, we detected F4/80 expression by immunohistochemistry. As shown in Figure 5. In the Sham group, there were only a few positive staining in lung sections. Remarkable strong staining was observed in the rats subjected to I/R, and the expression of F4/80 in the I/R group, DEX group, DA group, AI group, DW group, and WI group was higher than that in the Sham group (*P* < 0.05). In contrast, treatment with DEX decreased the expression of F4/80 compared to that of the I/R rats (*P* < 0.05). Treatment with AM630 or wortmannin increased the expressions of F4/80 compared to that of the DEX-treated rats (*P* < 0.05). AM630 alone or wortmannin alone had no significant impact on the expressions of F4/80 compared to that of the I/R rats (*P* > 0.05). This suggests that treatment with DEX may inhibit macrophage infiltration.

**Figure 6:**
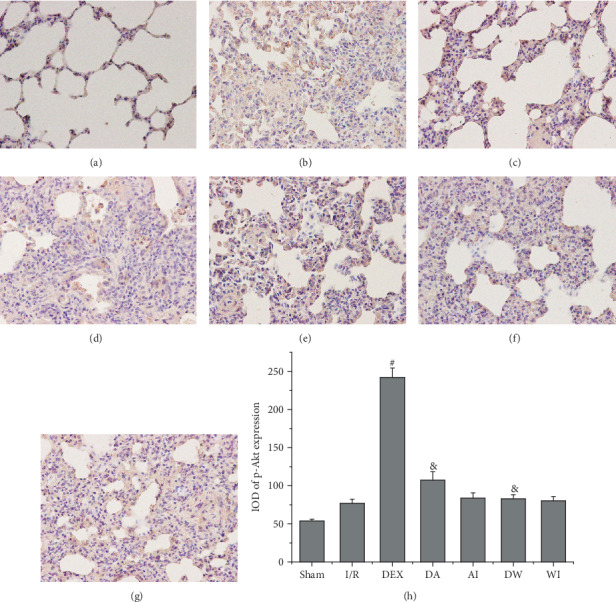
Immunohistochemical analysis of the expression of p-Akt in all groups. (a) Sham group. (b) Intestinal ischemia/reperfusion (I/R) group. (c) Dexmedetomidine-treated I/R (DEX) group. (d) Dexmedetomidine- and AM630-treated I/R (DA) group. (e) AM630-treated I/R (AI) group. (f) Dexmedetomidine- and wortmannin-treated I/R (DW) group. (g) Wortmannin-treated I/R (WI) group (original magnification, ×400). (h) Integrated optical density (IOD) of p-Akt expression in all groups. Data are expressed as mean ± SEM, *n* = 6 rats per group. ^∗^*P* < 0.05 versus the Sham group, ^#^*P* < 0.05 versus the I/R group, ^&^*P* < 0.05 versus the DEX group.

The lung tissues were obtained to measure the expression of p-Akt by immunohistochemistry, as shown in Figure 6. The results of immunohistochemistry showed that the level of p-Akt expression was low in the Sham group. I/R increased p-Akt expression, but the differences in the values between the Sham and I/R groups were not statistically significant (*P* > 0.05). Compared with the I/R group, the expression of p-Akt was enhanced in the DEX group (*P* < 0.05). The increase of p-Akt expression caused by DEX treatment was reversed by treatment with AM630 or wortmannin (*P* < 0.05). Compared with the I/R group, AM630 alone or wortmannin alone had no significant impact on p-Akt expression (*P* > 0.05).

### 3.7. The Effects of Dexmedetomidine Treatment on the Expressions of I/R Injury-Induced Apoptosis in Lung Tissues

**Figure 7:**
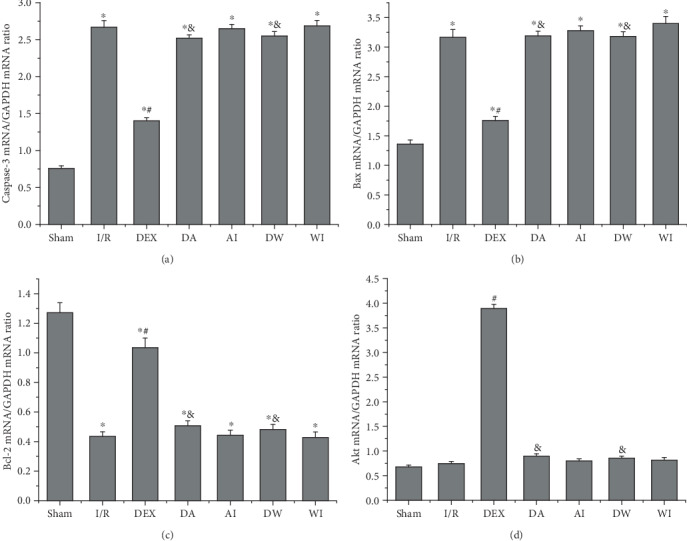
The effects of dexmedetomidine treatment on the mRNA expressions of caspase-3, Bax, Bcl-2, and Akt in all groups. (a) Caspase-3 mRNA expression. (b) Bax mRNA expression. (c) Bcl-2 mRNA expression. (d) Akt mRNA expression. Data are expressed as mean ± SEM, *n* = 6 rats per group. ^∗^*P* < 0.05 versus the Sham group, ^#^*P* < 0.05 versus the I/R group, ^&^*P* < 0.05 versus the DEX group. I/R: intestinal ischemia/reperfusion; DEX: dexmedetomidine-treated I/R; DA: dexmedetomidine- and AM630-treated I/R; AI: AM630-treated I/R; DW: dexmedetomidine- and wortmannin-treated I/R; WI: wortmannin-treated I/R.

**Figure 8:**
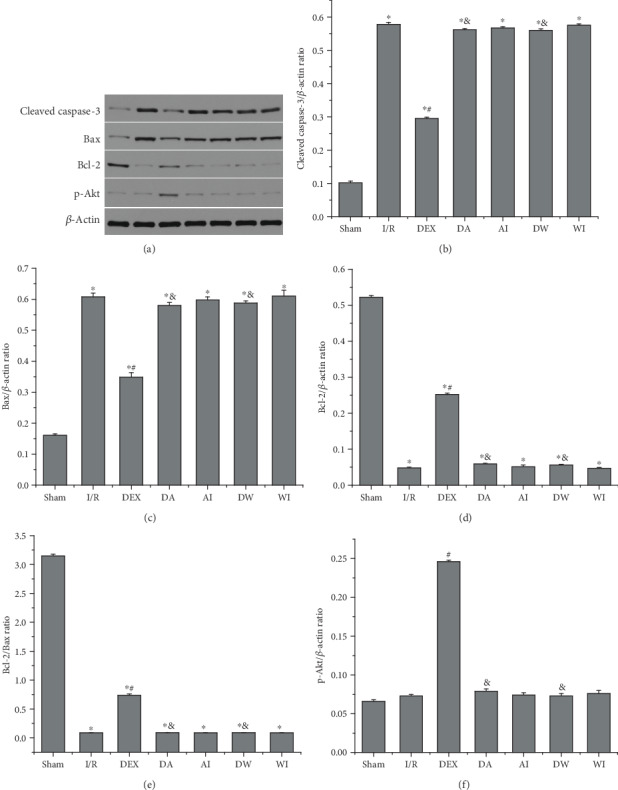
The effects of dexmedetomidine treatment on the protein expressions of cleaved caspase-3, Bax, Bcl-2, and p-Akt in all groups. (a) The expressions of cleaved caspase-3, Bax, Bcl-2, and p-Akt in lung tissues were detected using Western blot analysis. (b) Cleaved caspase-3 expression. (c) Bax expression. (d) Bcl-2 expression. (e) Bcl-2/Bax ratio. (f) p-Akt expression. Data are expressed as mean ± SEM, *n* = 6 rats per group. ^∗^*P* < 0.05 versus the Sham group, ^#^*P* < 0.05 versus the I/R group, ^&^*P* < 0.05 versus the DEX group. I/R: intestinal ischemia/reperfusion; DEX: dexmedetomidine-treated I/R; DA: dexmedetomidine- and AM630-treated I/R; AI: AM630-treated I/R; DW: dexmedetomidine- and wortmannin-treated I/R; WI: wortmannin-treated I/R.

Cell apoptosis is one of the main causes of lung injury caused by intestinal I/R. To investigate the role of cell apoptosis in lung injury caused by intestinal I/R, we evaluated the expressions of proapoptotic and antiapoptotic mRNA and proteins in lung tissues using RT-PCR and Western blot analysis. As shown in Figures 7(a)–7(c) and 8(a)–8(e), I/R upregulated the expressions of caspase-3 and Bax mRNA and cleaved caspase-3 and Bax proteins but reduced the expressions of Bcl-2 mRNA and protein (*P* < 0.05). The Bcl-2/Bax ratio was decreased significantly in the I/R rats (*P* < 0.05). Compared with the I/R group, the expressions of caspase-3 and Bax mRNA and cleaved caspase-3 and Bax proteins were downregulated significantly, the expressions of Bcl-2 mRNA and protein were increased, and the Bcl-2/Bax ratio was enhanced in the DEX group (*P* < 0.05). Compared with the DEX group, the expressions of caspase-3 and Bax mRNA and cleaved caspase-3 and Bax proteins were increased significantly, the expressions of Bcl-2 mRNA and protein were decreased, and the Bcl-2/Bax ratio was reduced in the rats given AM630 or wortmannin (*P* < 0.05). AM630 alone or wortmannin alone had no significant impact on the expressions of caspase-3 mRNA and cleaved caspase-3 protein, Bcl-2, and Bax mRNA and proteins compared to those of the I/R rats (*P* > 0.05).

### 3.8. The Effects of Dexmedetomidine Treatment on the Expressions of the PI3K/Akt Pathway

**Figure 9:**
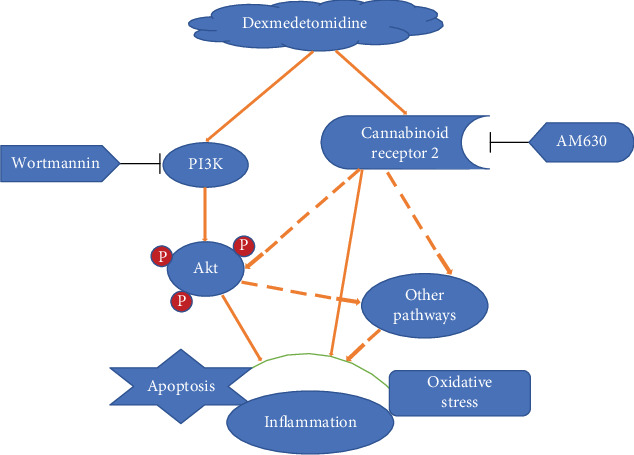
Schematic depicting model of potential molecular mechanism of protection of intestinal I/R-induced lung injury by dexmedetomidine. Dexmedetomidine may confer a protective role against intestinal I/R-induced lung injury by upregulating the CB_2_ receptor expression or activating the PI3K/Akt pathway. Treatment with the CB_2_ receptor antagonist, AM630, or the PI3K inhibitor, wortmannin, reverses the protective role of DEX by increasing the levels of apoptosis, inflammation, and oxidative stress (as marked by solid arrows). In addition, there may be a potential interaction between CB_2_ receptor and the PI3K/Akt pathway. CB2 receptor upregulated by dexmedetomidine may also play a protective role by activating Akt (or other pathways), because treatment with AM630 reduces Akt activation (as shown by dotted lines).

To investigate the protective mechanism of DEX on lung injury caused by intestinal I/R, we evaluated the expressions of Akt mRNA and proteins in lung tissues by RT-PCR and Western blot analysis. As shown in Figures 7(d), 8(a), and 8(f), I/R increased only the expressions of Akt mRNA and p-Akt protein, but there was no statistical difference, compared to those of the sham-operating rats (*P* > 0.05). Treatment with DEX increased the expressions of Akt mRNA and p-Akt protein compared to those of the I/R rats (*P* < 0.05). Treatment with AM630 or wortmannin downregulated the expressions of Akt mRNA and p-Akt protein compared to those of the DEX-treated rats (*P* < 0.05). AM630 alone or wortmannin alone had no significant impact on the expressions of Akt mRNA and p-Akt protein compared to those of the I/R rats (*P* > 0.05). These results suggest that the PI3K/Akt pathway may be involved in the protective role of CB_2_ receptor-mediated DEX treatment of I/R-induced lung injury. Collectively, our findings suggest that dexmedetomidine ameliorates lung injury induced by intestinal ischemia/reperfusion by upregulating the CB2 receptor expression or activating the PI3K/Akt pathway or CB2-mediated PI3K/Akt pathway (Figure 9).

## 4. Discussion

Intestinal I/R may not only cause local intestinal injury but also induce remote lung injury. In this study, we recognized that intestinal I/R led to lung injury, manifested by an increase in lung injury scores and an increased lung wet/dry ratio. At the same time, our results showed that dexmedetomidine attenuated lung injury by upregulating the CB_2_ receptor expression. However, the protective effects of dexmedetomidine treatment on lung injury induced by intestinal I/R were reversed by the CB_2_ receptor antagonist AM630. We also found that the CB_2_ receptor-mediated protective role of dexmedetomidine treatment of lung injury was achieved by activating the PI3K/Akt pathway.

Studies have suggested that oxidative stress is one of the major factors contributing to I/R injury [27]. In this study, we investigated and found that intestinal I/R resulted in an increase in MDA level and a decrease in SOD activity in the lung, aggravating lung tissue injury manifested by the increase in lung injury scores and lung wet/dry ratio. MDA, an end product of lipid peroxidation, is used to assess tissue peroxidative injury, while SOD, an antioxidant enzyme, is used to evaluate the antioxidant level of tissues. Several studies have indicated that I/R often increases the level of MDA and decreases the activity of SOD, leading to the aggravation of tissue damage [12, 25, 26, 28]. Our findings are consistent with those of these studies. Also, some therapeutic methods could reduce tissue damage by decreasing MDA levels and increasing SOD activity, such as antioxidant enzymes [25, 29, 30], and preconditioning [12].

Dexmedetomidine reportedly has an antioxidant effect, providing a protective role against damage in some tissues [7–12]. Here, we found that dexmedetomidine treatment alleviated lung injury caused by intestinal I/R via the inhibition of increased MDA levels and decreased SOD activity. Our findings are consistent with previous studies suggesting that the inhibition of lipid peroxidation and enhancement of antioxidant enzyme activity in the lungs might, at least, be partially involved in the protective mechanisms of dexmedetomidine in response to lung injury caused by intestinal I/R.

Inflammation and cell apoptosis play significant roles in the progression of lung injury caused by various factors, including I/R and shock. Some studies have indicated that pretreatment with some substances, such as cyclic arginine-glycine-aspartate peptide (cRGD) [31] and anti-osteopontin Ab [32], protects lung injury caused by intestinal I/R, lowers neutrophil infiltration, suppresses inflammation, and inhibits lung apoptosis after intestinal I/R. Ben et al. [33] established that lungs from TLR4 mutant mice undergoing intestinal I/R exhibited a marked reduction in epithelial apoptosis, pulmonary MPO activity, and TNF-*α* and IL-6 levels and manifested a significantly smaller histological injury. In this present study, intestinal I/R increased neutrophil and macrophage infiltration, pulmonary MPO activity, the levels of TNF-*α* and IL-6, and cell apoptosis, manifested by the increase in the expressions of caspase-3 mRNA and cleaved caspase-3 protein and decrease in the Bcl-2/Bax ratio that aggravated intestinal I/R-induced lung injury. In contrast, dexmedetomidine treatment reversed these changes, attenuating lung injury scores and lung wet/dry ratio and alleviating lung injury caused by intestinal I/R. Our results are, hence, consistent with these previous findings.

Although dexmedetomidine treatment has a protective role against intestinal I/R-induced lung injury, the exact mechanism is not entirely clear. Recent research has revealed a critical role for the CB_2_ receptor in regulating many disease processes, including in the immune system, cardiovascular and respiratory systems, bone, gastrointestinal tract and liver, and reproductive system [17, 34]. The CB_2_ receptor has also been verified to represent a very promising therapeutic target in gastrointestinal inflammatory conditions [18, 35]. Although the activation of the CB_2_ receptor may have positive symptomatic effects on inflammatory bowel disease through its anti-inflammatory effects [36], it is not clear whether the CB_2_ receptor is involved in the process of intestinal I/R-induced lung injury. In this study, our investigation of the role of the CB_2_ receptor in the lung injury caused by intestinal I/R showed that treatment with dexmedetomidine increased the expression of the CB_2_ receptor and attenuated lung injury, manifested by the decrease in lung injury scores and wet/dry ratio. Furthermore, the protective effect of dexmedetomidine treatment on lung injury caused by intestinal I/R was reversed by the CB_2_ receptor antagonist, AM630. Our results suggest that dexmedetomidine treatment provided the protective role against intestinal I/R-lung injury by activating the CB_2_ receptor. The role of the CB_2_ receptor in the I/R injuries of some other organs has also been demonstrated, including in the myocardium [37, 38], liver [22, 39], and brain [40]. Our findings are, therefore, consistent with these studies.

The PI3K/Akt is known as an intracellular signaling pathway and has been confirmed to provide a protective role against I/R injury by regulating cell proliferation, apoptosis, and oxidative stress [41]. PI3K/Akt activation is reportedly involved in the protective effect of DEX against the I/R injuries in some organs, including the lung [42, 43], heart [44], and kidney [45]. In our study, we also found that DEX treatment increased the expressions of Akt mRNA, p-Akt protein, and Bcl-2 mRNA and protein, reduced the expressions of caspase-3 mRNA, cleaved caspase-3 protein, and levels of TNF-*α*, IL-6, and MDA, and attenuated lung injury scores. However, when given the PI3K inhibitor wortmannin, the above protective effects of DEX were reversed, suggesting that DEX reduced intestinal I/R-induced lung injury by activating the PI3K/Akt pathway, consistent with earlier studies.

Also, some studies have revealed that the activation of the PI3K/Akt pathway plays a part in the protective mechanism of cannabinoid receptors. Both CB_1_ and CB_2_ receptors stimulate the proliferation of neural stem/precursor cells through the PI3K/Akt pathway [46]. Previous research showed that the CB_2_ receptor agonist, JWH133, protected the heart against I/R-induced apoptosis through the involvement of the PI3K/Akt pathway [47]. However, it is not clear whether the PI3K/Akt pathway mediates the protective effect of DEX activating the CB2 receptor. Here, DEX upregulated the expressions of Akt mRNA and p-Akt protein, attenuated lung injury scores, and reduced intestinal I/R-induced lung injury. In contrast, the use of both the CB_2_ receptor antagonist, AM630, and the PI3K inhibitor, wortmannin, reversed the protective roles of DEX against lung injury. Additionally, the CB_2_ receptor antagonist AM630 downregulated the expression of Akt mRNA and p-Akt protein and increased lung injury scores. Our results indicate that DEX protected against lung injury caused by intestinal I/R via the CB_2_-mediated PI3K/Akt pathway.

The present study has some limitations. Although we investigated that the protective roles of DEX against lung injury caused by intestinal I/R were reversed by both the CB_2_ receptor antagonist, AM630, and the PI3K inhibitor, wortmannin, there was insufficient evidence to draw the conclusion that DEX treatment provides a protective role against intestinal I/R-induced lung injury through the activation of the CB_2_-mediated PI3K/Akt pathway. Further study using deficient animals is needed.

## 5. Conclusions

Our data show that DEX treatment provided a protective effect on lung injury caused by intestinal I/R, with the protective effects attributed mainly to the upregulation of the CB_2_ receptor and subsequent activation of the PI3K/Akt pathway. The current findings provide a basis for the development of novel strategies for the treatment of lung injury.

## Data Availability

The authors have authorised me to promise that the data in our manuscript can be provided to anyone who needs it. If anyone needs these data, please contact me by the email hexh1220@aliyun.com.

## References

[1] Mallick I. H., Yang W., Winslet M. C., Seifalian A. M. (2004). Ischemia-reperfusion injury of the intestine and protective strategies against injury. *Digestive Diseases and Sciences*.

[2] An S., Hishikawa Y., Liu J., Koji T. (2007). Lung injury after ischemia-reperfusion of small intestine in rats involves apoptosis of type II alveolar epithelial cells mediated by TNF-*α* and activation of Bid pathway. *Apoptosis*.

[3] Hill J., Lindsay T. F., Ortiz F., Yeh C. G., Hechtman H. B., Moore F. D. (1992). Soluble complement receptor type 1 ameliorates the local and remote organ injury after intestinal ischemia-reperfusion in the rat. *Journal of Immunology*.

[4] Sorkine P., Setton A., Halpern P. (1995). Soluble tumor necrosis factor receptors reduce bowel ischemia-induced lung permeability and neutrophil sequestration. *Critical Care Medicine*.

[5] de Perrot M., Liu M., Waddell T. K., Keshavjee S. (2003). Ischemia-reperfusion-induced lung injury. *American Journal of Respiratory and Critical Care Medicine*.

[6] Cai Y., Xu H., Yan J., Zhang L., Lu Y. (2014). Molecular targets and mechanism of action of dexmedetomidine in treatment of ischemia/reperfusion injury (Review). *Molecular Medicine Reports*.

[7] Chen Z., Ding T., Ma C. G. (2017). Dexmedetomidine (DEX) protects against hepatic ischemia/reperfusion (I/R) injury by suppressing inflammation and oxidative stress in NLRC5 deficient mice. *Biochemical and Biophysical Research Communications*.

[8] Tüfek A., Tokgöz O., Aliosmanoglu I. (2013). The protective effects of dexmedetomidine on the liver and remote organs against hepatic ischemia reperfusion injury in rats. *International Journal of Surgery*.

[9] Jiang L., Li L., Shen J., Qi Z., Guo L. (2014). Effect of dexmedetomidine on lung ischemia-reperfusion injury. *Molecular Medicine Reports*.

[10] Yoshikawa Y., Hirata N., Kawaguchi R., Tokinaga Y., Yamakage M. (2018). Dexmedetomidine maintains its direct cardioprotective effect against ischemia/reperfusion injury in hypertensive hypertrophied myocardium. *Anesthesia and Analgesia*.

[11] Li Q., Chen C., Chen X., Han M., Li J. (2018). Dexmedetomidine attenuates renal fibrosis via *α*2-adrenergic receptor-dependent inhibition of cellular senescence after renal ischemia/reperfusion. *Life Sciences*.

[12] Zhang X. Y., Liu Z. M., Wen S. H. (2012). Dexmedetomidine administration before, but not after, ischemia attenuates intestinal injury induced by intestinal ischemia-reperfusion in rats. *Anesthesiology*.

[13] Li J., Chen Q., He X. (2018). Dexmedetomidine attenuates lung apoptosis induced by renal ischemia-reperfusion injury through *α*_2_AR/PI3K/Akt pathway. *Journal of Translational Medicine*.

[14] Zhang J. J., Peng K., Zhang J., Meng X. W., Ji F. H. (2017). Dexmedetomidine preconditioning may attenuate myocardial ischemia/reperfusion injury by down-regulating the HMGB_1_-TLR_4_-MyD88-NF-*к*B signaling pathway. *PLoS One*.

[15] Si Y., Bao H., Han L. (2013). Dexmedetomidine protects against renal ischemia and reperfusion injury by inhibiting the JAK/STAT signaling activation. *Journal of Translational Medicine*.

[16] Shen J., Fu G., Jiang L., Xu J., Li L., Fu G. (2013). Effect of dexmedetomidine pretreatment on lung injury following intestinal ischemia-reperfusion. *Experimental and Therapeutic Medicine*.

[17] Patel K. D., Davison J. S., Pittman Q. J., Sharkey K. A. (2010). Cannabinoid CB(2) receptors in health and disease. *Current Medicinal Chemistry*.

[18] Wright K. L., Duncan M., Sharkey K. A. (2008). Cannabinoid CB2 receptors in the gastrointestinal tract: a regulatory system in states of inflammation. *British Journal of Pharmacology*.

[19] Alexander A., Smith P. F., Rosengren R. J. (2009). Cannabinoids in the treatment of cancer. *Cancer Letters*.

[20] Dhopeshwarkar A., Mackie K. (2014). CB2 cannabinoid receptors as a therapeutic target-what does the future hold?. *Molecular Pharmacology*.

[21] Pacher P., Haskó G. (2008). Endocannabinoids and cannabinoid receptors in ischaemia-reperfusion injury and preconditioning. *British Journal of Pharmacology*.

[22] Horváth B., Magid L., Mukhopadhyay P. (2012). A new cannabinoid CB2 receptor agonist HU-910 attenuates oxidative stress, inflammation and cell death associated with hepatic ischaemia/reperfusion injury. *British Journal of Pharmacology*.

[23] Defer N., Wan J., Souktani R. (2009). The cannabinoid receptor type 2 promotes cardiac myocyte and fibroblast survival and protects against ischemia/reperfusion-induced cardiomyopathy. *The FASEB Journal*.

[24] Lehmann C., Kianian M., Zhou J. (2012). Cannabinoid receptor 2 activation reduces intestinal leukocyte recruitment and systemic inflammatory mediator release in acute experimental sepsis. *Critical Care*.

[25] He X. H., Yan X. T., Wang Y. L., Wang C. Y., Zhang Z. Z., Zhan J. (2014). Transduced PEP-1–heme oxygenase-1 fusion protein protects against intestinal ischemia/reperfusion injury. *The Journal of Surgical Research*.

[26] He X. H., Li Q. W., Wang Y. L. (2015). Transduced PEP-1-heme oxygenase-1 fusion protein reduces remote organ injury induced by intestinal ischemia/reperfusion. *Medical Science Monitor*.

[27] Ferrari R. S., Andrade C. F. (2015). Oxidative stress and lung ischemia-reperfusion injury. *Oxidative Medicine and Cellular Longevity*.

[28] Melo L. G., Agrawal R., Zhang L. (2002). Gene therapy strategy for long-term myocardial protection using adeno-associated virus-mediated delivery of heme oxygenase gene. *Circulation*.

[29] Zhang Y. E., Wang J. N., Tang J. M. (2009). *In vivo* protein transduction: delivery of PEP-_1_-SOD_1_ fusion protein into myocardium efficiently protects against ischemic insult. *Molecules and Cells*.

[30] Huang G. Q., Wang J. N., Tang J. M. (2011). The combined transduction of copper, zinc-superoxide dismutase and catalase mediated by cell-penetrating peptide, PEP-1, to protect myocardium from ischemia-reperfusion injury. *Journal of Translational Medicine*.

[31] Matsuo S., Yang W. L., Aziz M., Jacob A., Wang P. (2013). Cyclic arginine-glycine-aspartate attenuates acute lung injury in mice after intestinal ischemia/reperfusion. *Critical Care*.

[32] Hirano Y., Aziz M., Yang W. L., Ochani M., Wang P. (2016). Neutralization of osteopontin ameliorates acute lung injury induced by intestinal ischemia-reperfusion. *Shock*.

[33] Ben D. F., Yu X. Y., Ji G. Y. (2012). TLR4 mediates lung injury and inflammation in intestinal ischemia-reperfusion. *The Journal of Surgical Research*.

[34] Chen D. J., Gao M., Gao F. F., Su Q. X., Wu J. (2017). Brain cannabinoid receptor 2: expression, function and modulation. *Acta Pharmacologica Sinica*.

[35] Izzo A. A. (2007). The cannabinoid CB (2) receptor: a good friend in the gut. *Neurogastroenterology and Motility*.

[36] Leinwand K. L., Jones A. A., Huang R. H. (2017). Cannabinoid receptor-2 ameliorates inflammation in murine model of Crohn's disease. *Journal of Crohn's & Colitis*.

[37] Sun H. J., Lu Y., Wang H. W. (2017). Activation of endocannabinoid receptor 2 as a mechanism of propofol pretreatment-induced cardioprotection against ischemia-reperfusion injury in rats. *Oxidative Medicine and Cellular Longevity*.

[38] Wang P. F., Jiang L. S., Bu J. (2012). Cannabinoid-2 receptor activation protects against infarct and ischemia-reperfusion heart injury. *Journal of Cardiovascular Pharmacology*.

[39] Rajesh M., Pan H., Mukhopadhyay P. (2007). Cannabinoid-2 receptor agonist HU-308 protects against hepatic ischemia/reperfusion injury by attenuating oxidative stress, inflammatory response, and apoptosis. *Journal of Leukocyte Biology*.

[40] Zhang M., Adler M. W., Abood M. E., Ganea D., Jallo J., Tuma R. F. (2009). CB2 receptor activation attenuates microcirculatory dysfunction during cerebral ischemic/reperfusion injury. *Microvascular Research*.

[41] Williams D. L., Ozment-Skelton T., Li C. (2006). Modulation of the phosphoinositide 3-kinase signaling pathway alters host response to sepsis, inflammation, and ischemia/reperfusion injury. *Shock*.

[42] Liang S., Wang Y., Liu Y. (2019). Dexmedetomidine alleviates lung ischemia-reperfusion injury in rats by activating PI3K/Akt pathway. *European Review for Medical and Pharmacological Sciences*.

[43] Zhang W., Zhang J. Q., Meng F. M., Xue F. S. (2016). Dexmedetomidine protects against lung ischemia-reperfusion injury by the PI3K/Akt/HIF-1*α* signaling pathway. *Journal of Anesthesia*.

[44] Cheng X., Hu J., Wang Y. (2018). Effects of dexmedetomidine postconditioning on myocardial ischemia/reperfusion injury in diabetic rats: role of the PI3K/Akt-dependent signaling pathway. *Journal Diabetes Research*.

[45] Gu J., Sun P., Zhao H. (2011). Dexmedetomidine provides renoprotection against ischemia-reperfusion injury in mice. *Critical Care*.

[46] Molina-Holgado F., Rubio-Araiz A., García-Ovejero D. (2007). CB2 cannabinoid receptors promote mouse neural stem cell proliferation. *The European Journal of Neuroscience*.

[47] Li Q., Wang F., Zhang Y. M., Zhou J. J., Zhang Y. (2013). Activation of cannabinoid type 2 receptor by JWH133 protects heart against ischemia/reperfusion-induced apoptosis. *Cellular Physiology and Biochemistry*.

